# Sustainable conversion of coffee and other crop wastes to biofuels and bioproducts using coupled biochemical and thermochemical processes in a multi-stage biorefinery concept

**DOI:** 10.1007/s00253-014-5991-1

**Published:** 2014-09-11

**Authors:** Stephen R. Hughes, Juan Carlos López-Núñez, Marjorie A. Jones, Bryan R. Moser, Elby J. Cox, Mitch Lindquist, Luz Ángela Galindo-Leva, Néstor M. Riaño-Herrera, Nelson Rodriguez-Valencia, Fernando Gast, David L. Cedeño, Ken Tasaki, Robert C. Brown, Al Darzins, Lane Brunner

**Affiliations:** 1 Agricultural Research Service (ARS), National Center for Agricultural Utilization Research (NCAUR), Renewable Product Technology (RPT) Research Unit, United States Department of Agriculture (USDA), 1815 North University Street, Peoria, IL 61604 USA; 2 National Coffee Research Centre (Cenicafe), National Federation of Coffee Growers of Colombia (FNC), Cenicafé Planalto Km 4 vía Antigua Chinchiná, Manizales, Caldas Colombia; 3 Department of Chemistry, Illinois State University, 214 Julian Hall 4160, Normal, IL 61790-4160 USA; 4 Agricultural Research Service (ARS), National Center for Agricultural Utilization Research (NCAUR), Bio-Oils Research (BOR) Unit, United States Department of Agriculture (USDA), 1815 North University Street, Peoria, IL 61604 USA; 5 USMC Research and Innovation, Mitsubishi Chemical, 410 Palos Verdes Blvd, Redondo Beach, CA 90277 USA; 6 1140 E Biorenewables Research Laboratory, Iowa State University, Ames, IA 50011 USA; 7 Gas Technology Institute, 1700 S Mount Prospect Road, Des Plaines, IL 60018 USA; 8 Gen2 Energy, 276 Main St, Farmington, CT 06032 USA

**Keywords:** Coffee waste, Multi-stage biorefinery, Oleaginous yeast triglycerides, Renewable biofuel, Bioprocessing

## Abstract

The environmental impact of agricultural waste from the processing of food and feed crops is an increasing concern worldwide. Concerted efforts are underway to develop sustainable practices for the disposal of residues from the processing of such crops as coffee, sugarcane, or corn. Coffee is crucial to the economies of many countries because its cultivation, processing, trading, and marketing provide employment for millions of people. In coffee-producing countries, improved technology for treatment of the significant amounts of coffee waste is critical to prevent ecological damage. This mini-review discusses a multi-stage biorefinery concept with the potential to convert waste produced at crop processing operations, such as coffee pulping stations, to valuable biofuels and bioproducts using biochemical and thermochemical conversion technologies. The initial bioconversion stage uses a mutant *Kluyveromyces marxianus* yeast strain to produce bioethanol from sugars. The resulting sugar-depleted solids (mostly protein) can be used in a second stage by the oleaginous yeast *Yarrowia lipolytica* to produce bio-based ammonia for fertilizer and are further degraded by *Y. lipolytica* proteases to peptides and free amino acids for animal feed. The lignocellulosic fraction can be ground and treated to release sugars for fermentation in a third stage by a recombinant cellulosic *Saccharomyces cerevisiae*, which can also be engineered to express valuable peptide products. The residual protein and lignin solids can be jet cooked and passed to a fourth-stage fermenter where *Rhodotorula glutinis* converts methane into isoprenoid intermediates. The residues can be combined and transferred into pyrocracking and hydroformylation reactions to convert ammonia, protein, isoprenes, lignins, and oils into renewable gas. Any remaining waste can be thermoconverted to biochar as a humus soil enhancer. The integration of multiple technologies for treatment of coffee waste has the potential to contribute to economic and environmental sustainability.

## Introduction

### Economic importance of coffee

Coffee is said to be the second most traded commodity in the world after petroleum, signifying its importance to the global economy (ACDI/VOCA 2014, Beelarts 2011). More than 2.3 billion cups of coffee are consumed in the world every day. Most consumption takes place in industrialized countries, while over 90 % of coffee production occurs in developing countries. For example, in Brazil, the world’s largest coffee producer, over five million people are involved in the cultivation and harvesting of coffee plants. (Beelarts 2011). It is crucial to the economies and politics of many developing countries because its cultivation, processing, trading, and marketing provide employment for millions of people (Mussatto et al. 2011). Colombia was the third largest coffee producer in the world after Brazil and Vietnam until 2009 when it was overtaken by Indonesia. In 2013, Brazil produced 56.1 million 60-kg bags of green coffee, Vietnam 26.5 million, Indonesia 10.5 million, and Colombia 9.9 million (Index mundi 2014).

### Role of coffee in Colombia

Coffee is Colombia’s main agricultural export (FAO 2013). Coffee cultivation and export are under the management of the Federación Nacional de Cafeteros (FNC; National Coffee Growers Federation), which is the country’s most important private enterprise. The federation sponsors farmers in the coffee-growing zones through extensive social and economic aid programs. Colombian coffee is the name given to a 100 % washed Arabica coffee produced in the coffee-growing regions of Colombia (FNC-1 2010). It originates from the particular combination of diverse factors: the latitude and altitude of Colombia’s coffee growing zone, its soils, the botanical origin of the species, and the varieties of coffees produced, the climate and rain pattern generated by the double path of the Intertropical Convergence Zone over the coffee area, the ever changing topography, the luminosity, the favorable temperature range within the day and throughout the year, an adequate amount and distribution of the rain, and some common cultural practices that include the processes of selective harvesting and of transformation of the fruit through its washing and drying (FNC-1 2010). Furthermore, the tradition of the selective harvesting of Colombian coffee, the variety, the wet processing, the drying process and its subsequent classification and threshing are also the basis for the optimum quality of the product (Alvarado et al. 2002; FNC-1 2010). The production of green (unroasted) coffee beans by year in Colombia over the past decade is shown in Table 1.

**Table 1:** Green coffee production in Colombia from 2004 to 2013 (reference: Index mundi 2014)

Market year	Production (million 60-kg bags)	Growth rate (%)
2004	11.1	−5.6
2005	11.5	4.3
2006	12.0	3.7
2007	12.2	1.8
2008	12.5	2.9
2009	8.7	−30.8
2010	8.1	−6.5
2011	8.5	5.3
2012	7.7	−10.2
2013	9.9	29.7

### Environmental impact of coffee production

No matter where coffee is grown, discharge from coffee processing plants represents a major source of river pollution in Central and South America. The process of separating the commercial product (the bean) from coffee cherries generates enormous volumes of waste material in the form of pulp, residual water, and parchment. For example, the Guatemala-based Instituto Centroamericano de Investigación y Tecnología Industrial estimated that over a 6-month period during 1988, the processing of 547,000 tons of coffee in Central America generated 1.1 million tons of pulp and polluted 110,000 m^3^ of water per day, resulting in discharge into the region’s waterways equivalent to raw sewage from a city of 4 million people (NRDC 2014). It has been calculated that processing of every one million 60-kg bags of dried coffee beans produces 218,400 tons of fresh pulp and mucilage, which has a chemical oxygen demand equivalent to that of the sewage generated in 1 year by 1.2 million people (Veenstra 1995). The FNC’s National Coffee Research Center (Cenicafé) in Colombia has conducted research on coffee production, harvesting methods, wet mill processes, quality, by-products management, and natural resource conservation. The protection, recovery, and appropriate management of soils and water sources are a priority in Colombia, and Cenicafé has been recognized for their efforts in promoting water conservation, prevention and control of water pollution, and sustainable practices for the use of water sources (Cenicafé 2011).

## Colombian coffee production

### Coffee cultivation in Colombia

The Arabica coffee produced in Colombia needs specific climatic conditions for its production. The ideal conditions for the cultivation of this species are found between 1,200 m (4,000 ft) and 1,800 m (6,000 ft) above sea level, with temperatures between 17 and 23 °C (62 and 75 °F), and with precipitation close to 2,000 mm (78 in.) per year, evenly distributed throughout the year. The specific geographic location of each Colombian coffee growing region determines its particular conditions of water availability, temperature, solar radiation, and wind regime for coffee cultivation (FNC-2 2010). Most Colombian coffee growing areas are located in the Colombian Departments of Antioquia, Boyacá, Caldas, Cauca, Cesar, Caquetá, Casanare, Cundinamarca, Guajira, Huila, Magdalena, Meta, Nariño, Norte de Santander, Quindío, Risaralda, Santander, Tolima, and Valle. In most of the coffee-growing regions in the country, there is a period of flowering from January to March, and another one from July to September. The main harvest in these zones takes place between September and December, and there is a secondary harvest during the second quarter of the year (FNC-2 2010).

The mature coffee bean is either red or yellow in color. Each bean has an exterior skin (exocarp) that wraps around a sweet pulp-like substance (mesocarp). Under the pulp are beans covered by a delicate and translucent membrane (silver skin) and these layers sheath the two internal coffee seeds (endosperm). These seeds are roasted in order to produce the beverage that consumers recognize as “coffee.” The post-harvest processes, commonly referred to as the “beneficio” serve to transform the coffee cherry into a dry product ready to be roasted.

The wet beneficio process includes de-pulping, fermentation, washing, and drying the coffee bean. In the first step, the coffee bean is de-pulped immediately after being harvested. Subsequently, the mucilage is removed mechanically or by means of hydraulic fermentation. Because the time needed to ferment the coffee beans is a critical factor in the quality of the coffee, samples are taken periodically from the fermentation tanks to determine the optimal point to initiate the final washing process. After the fermentation process is completed, the beans are washed to eliminate the mucilage from the bean. The wet beneficio process is linked with the Colombian coffee tradition and constitutes one of the principal elements in guaranteeing the quality of coffee. Once the coffee has gone through the wet beneficio process, it is dried naturally through exposure to sunlight or in mechanical dryers. The dried coffee seeds are commonly referred to as parchment coffee. When the drying process is completed, the coffee undergoes a process referred to as hulling, which removes the parchment from the beans in order to obtain green coffee. The green coffee seeds are then selected and classified in terms of size, weight, color, and physical appearance (FNC-3 2010).

### Impact of Colombian coffee processing on water quality

During the wet method of processing coffee, enormous amounts of biowaste are generated in the form of pulp and residual water. This wastewater is high in organic matter and acidity content with a chemical oxygen demand (COD; a rapid measure of the total quantity of oxygen required to oxidize all organic material into carbon dioxide and water) value that varies between 18,000 and 30,000 mg per liter. The organic matter in agricultural waste runoff is decomposed by water-borne bacteria using dissolved oxygen. In cases of substantial discharge of wastewater into natural water bodies, the oxygen is significantly depleted, thereby destroying aquatic plants and animals (UTZ Certified 2013).

In 2004, the government of Colombia contracted with Resources for the Future (RFF), a nonprofit research organization, to study the effectiveness and efficiency of Colombia’s environmental policies (Blackman et al. 2006). The final report assesses those policies from 1993 to 2003 that address environmental planning and management, as well as those that utilize command-and-control regulations, market-based instruments, legal mechanisms, administrative procedures, and mediation. It identified areas of concern in the environmental management system. In examining water quality, it reported that according to the Institute of Hydrology, Meteorology and Environmental Studies (IDEAM), the collection and management of information on ambient water quality in Colombia were not adequate. Monitoring stations exist, but coverage was limited and data collection and management were not standardized. According to the RFF report, although reliable analysis of water pollution at the national level was not available, Colombia’s National Planning Department (DNP) estimated that in 1994, the three largest contributors to biochemical oxygen demand (the principal measure of organic pollutant discharges) in surface waters in Colombia were: (1) agricultural and livestock nonpoint sources (84 %); (2) domestic wastewater from large urban centers (10 %); and (3) industrial point sources (6 %). Typically, in most countries with significant agricultural sectors, nonpoint sources, which are particularly difficult to control, are the leading cause of water pollution. There is a general consensus that many of Colombia’s water basins are severely polluted, with the Bogotá, Cali, Cauca, Medellín, de Oro, Lebrija, Pasto, Pamplonita, Combeima, and Otún rivers are in critical condition (Blackman et al. 2006).

Although Colombia has abundant fresh water resources, water scarcity has become a problem in some regions. If management of watersheds is not improved, it is estimated that vulnerability to surface water shortages will grow and give rise to significant problems in the Andes and Caribbean regions. The use of pesticides and agro-chemicals in the coffee cultivation process has led to the contamination of water sources with nitrates, sulfates, and phosphates. Deforestation of the watersheds of the middle and upper basins of the Cauca and Magdalena Rivers has destabilized water sources balance and increased erosion in Colombia’s coffee region (Slunge 2008).

## Progress toward sustainable coffee production in Colombia

### Water pollution reduction in Colombia

Recent years have witnessed important progress in the development of pollution control technologies in coffee processing. Coffee pulping stations using the Ecomill technology (Colombian Coffee Growers 2013; Oliveros-Tascón et al. 2011) are substantially reducing the volume of water used in the wet processing of coffee; this, in turn, reduces the amount of water requiring treatment before being discharged from the processing facilities. Additional environmentally sound measures have been implemented, including composting coffee husks mixed with farm animal manure to use as organic fertilizer on crops and building anaerobic digesters that produce methane gas that can be used for powering the processing plant (NRDC 2014).

Environmental protection and sustainability are vital to the success of the coffee growing and production industry. As a result, in Colombia, much of the work of the FNC and Cenicafé is dedicated to understanding the relationship between coffee growing and the environment and finding techniques to minimize the environmental impact at each stage of coffee production (Colombian Coffee Growers 2013). In order to avoid the contamination of water resources with nitrates, sulfates, and phosphates, the research performed by Cenicafé has led to the reduced use of pesticides and agro-chemicals in the coffee cultivation process. The reduction was possible due to the renewal of coffee crops with Arabica varieties resistant to coffee rust, the implementation of integrated management of coffee pests and diseases, and the maintenance of the soil’s productive capacity and natural fertility. Another area of research is the stabilization of water sources balance and reduction of erosion in Colombia’s coffee region. The reforestation of the watersheds of the middle and upper basins of the Cauca and Magdalena Rivers, and the increase of sustainable forest practices and ecosystem protection are some of Cenicafé’s major initiatives. Furthermore, Cenicafé’s developments for decreasing water usage in the different stages of the wet processing of coffee have managed to substantially reduce water consumption (Colombian Coffee Growers 2013).

The most important technology developed by Cenicafé and currently available to Colombian coffee growers is the new Ecomill coffee washing technology, which significantly reduces water and energy consumption and completely eliminates wastewater contamination during the de-pulping or processing stages. It allows coffee to be washed by a process of natural fermentation (or by applying pectinolytic enzymes) using between 0.35 and 0.6 l of water per kilogram (L/kg) of dried parchment coffee (cps) produced. This level of water consumption is extremely low compared to washing in vats with manual agitation, which requires 4.2 L/kg cps, or washing in larger-sized tanks with waterproof pumps (requires between 6 and 9 L/kg cps) or using washing channels (requires 20 L/kg cps) (Oliveros-Tascón et al. 2011). The previous Becolsub technology, which was also developed by Cenicafé, had already reduced water consumption to between 0.7 and 1.0 L/kg cps. The Ecomill technology can be used to produce mild coffee not only with a significant reduction in water usage but also most notably with a 100 % reduction of the contamination generated by wastewater during the washing process. The environmental advances made by deploying Ecomill in comparison to earlier technologies are especially important in times when global environmental awareness is on the rise, as is the demand for sustainable agricultural products, including coffee (Colombian Coffee Growers 2013).

### Ongoing sustainability efforts in Colombia

Eco-certification of food and other agricultural products has been promoted as a way of making markets work for sustainability. Certification programs offer a price premium to producers who invest in more sustainable practices. Several certification programs exist today for global commodities such as timber, coffee, cocoa, and bananas with different claims to sustainability (Dauvergne and Lister 2012; Giovannucci and Ponte 2005). Four certification programs include the coffee sector: Rainforest Alliance, UTZ, Organic, and Fair Trade. The literature on the impacts of certification has focused primarily on the economic benefits farmers perceive from participating in these programs. The economic benefits, however, are often subject to price variability, offering only a partial explanation of why farmers join and stay in certification programs (Hughell and Newsom 2013).

Rueda and Lambin (2013) evaluated the potential of the Rainforest Alliance certification program to foster more resilient social–ecological systems in the face of globalization. Using the case of Santander, Colombia, and a pair-based comparison of 86 households, Rueda and Lambin showed that certification provides important environmental benefits, while improving the well-being of farmers and their communities. Furthermore, their study showed that price premiums are only one of many elements defining the success of certification, particularly important for motivating farmers to join, but less so to explain retention and upgrading. In fact, non-premium benefits explain retention in the certification program. Through certification, farmers acquired skills and abilities that helped them mobilize assets and make their operation more sustainable, even in the face of decreasing premiums. They also gained access to information, technology, social networks, and resources that were not able to reach them before they became certified. Coffee growers widened their access to market outlets whose prices are more stable and have done so while enhancing local agro-ecosystems. Small farmers, supported by a strong institutional arrangement that provides technical and commercial services, have not only coped with market trends but also adapted to changing conditions in the global economy to achieve more sustainable livelihoods and land use practices (Blackman et al. 2006; Rueda and Lambin 2013).

### Utilization of biomass waste materials in Colombia

Colombia is a major producer of agricultural and animal commodities (index mundi 2014), and these operations generate large amounts of residues and wastes. The agricultural residues and animal waste can be used to produce energy and other products in systems similar to an ethanol refinery where the production process involves conversion of biomass into fuel, energy, and chemicals, integrated in the context of a biorefinery. The main geographical regions generating biomass waste are the Magdalena Valley with palm oil, sugarcane, and other crops; the eastern slopes and the western mountain range in the vicinity of Bogota with its palm plantations; the Department of Antioquia and Valle del Cauca where coffee and sugarcane are produced; and in the regions where coffee pulping operations are located (Keesman 2011).

In addition to pulp and mucilage waste from coffee processing, Colombia has an abundance of biomass in the form of sugar cane. The rainfall patterns in the region near the Pacific Coast where the sugar is grown for the biofuels industry allows the crop to be harvested throughout the year. Sugar cane is also grown farther east, but because of constant rain from April to August, it can only be harvested 8 months out of the year. After the sugars are extracted, the remaining bagasse, which comprises a majority of the plant’s mass and gives it rigidity and structure, can be stacked and stored. Currently, bagasse is burned to produce steam that drives turbines for the generation of electricity. The Energy Department’s National Renewable Energy Laboratory (NREL) is working with Ecopetrol, the largest oil company in Colombia, to process the residue from sugar cane and palm oil harvesting into fuel ethanol for blending with gasoline (NREL 2013). The aim is to optimize the conversion process for bagasse and to analyze the economic case for commercial production of biofuel from these materials. It includes a study on palm rachis, the material left over after palm oil production, as an example. The hope is that commercial conversion facilities and employees can be kept in operation all year by processing the bagasse during the rainy season when it is too wet to get to the fields and harvest the sugar cane (NREL 2013).

In 2011, better weather conditions in the sugarcane area resulted in an increase in sugarcane supply for sugar and ethanol. Colombian palm oil and sugarcane production well exceeded the local demand and generated a surplus that sustained biofuels production. Ethanol production was 351 million liters, which was 25 % higher than the previous year. There are several studies in Colombia looking for financing to produce ethanol and biodiesel with feedstocks other than sugarcane and palm oil. Because Colombia’s biofuels capacity has not reached the initial biofuel blend mandated by the government, the Colombian government has allowed the amount of ethanol blended with biofuels to increase along with the increase in production as new facilities expand or enter into production (Global 2011).

Colombia is experienced in biofuel production through fermentation to produce ethanol and the transesterification process to produce biodiesel. The Ministry of Environment (MAVDT) is collaborating with the industry to further develop biomass in Colombia by involving the rural, agricultural, and energy sectors in an integrated effort of research and pilot projects to demonstrate the viability of biomass as an energy source. In addition to cellulosic ethanol, another option being considered is the gasification of biomass to obtain synthetic gas (syngas), subsequent purification of the gas, and generation of synthetic diesel and gasoline (Keesman 2011).

In Colombia, agriculture is one of the sectors most affected by climate change, and at the same time, the sector contributes the most to global warming. Climate change is causing a shift from coffee to corn (maize) production. Climate conditions, particularly changes in availability of water, affect animal and crop productivity. Colombia’s National Planning Department (DNP) is coordinating a project aimed at quantifying the economic cost of climate change impacts and determining optimal responses. Corn, rice, potatoes, and sugarcane were selected as the first crops to consider based on their economic and food security importance. The crops were matched to production regions, where information about soils, soil use, type of agricultural practices, and climate conditions was collected. The project identified climate scenarios and simulated growth and yields for each region under different climate scenarios. The results are helping farmers to identify adaptation measures in the types of crops they grow that can reduce hunger and improve nutrition (Loboguerrero and Vermeulen 2013).

## Potential conversion technologies for coffee and other agricultural wastes

### Coupled biochemical and thermochemical conversion technologies

Agricultural wastes from coffee operations are complex materials (Navia et al. 2011) as illustrated by an analysis of a mixture of 60 % pulp plus 40 % mucilage simulating coffee waste presented in Table 2. A combination of technologies will likely be required to convert waste produced at crop-processing operations, such as coffee pulping stations, to valuable biofuels and bioproducts. These might involve a series of microbial (biochemical) and thermochemical conversion processes as well as water treatment processes in an integrated biorefinery strategy. The initial bioconversion in the proposed biorefinery concept is fermentation of the sugars present in the waste by a thermotolerant mutant *Kluyveromyces marxianus* yeast strain, NRRL Y-50798 (Hughes et al. 2013). The resulting sugar-depleted solids (mostly protein) can subsequently be subjected to treatment by the oleaginous yeast *Yarrowia lipolytica* NRRL YB-567 var. F to produce bio-based ammonia for fertilizer. The residual protein can then be degraded by *Yarrowia lipolytica* proteases to peptides and free amino acids for animal feed. The lignocellulosic fraction of the waste can be ground and treated to release sugars for fermentation by a recombinant strain of *Saccharomyces cerevisiae* (Hughes et al. 2009b). *Saccharomyces cerevisiae* NRRL Y-50183 has the potential to be engineered to produce valuable peptide products in this step. In the last biochemical step, the residual solids including protein and lignin can be jet cooked and passed to a fermenter where an engineered mutagenized strain of *Rhodotorula glutinis* NRRL Y-12906 can convert the resulting materials and methane from anaerobic digestion operations into isoprenoid intermediates. The resulting fermentation residues can be combined and transferred into pyrocracking and hydroformylation reactions to convert ammonia, protein, isoprenoid intermediates, lignins, and oils into renewable gasoline. Any remaining waste can be thermoconverted to biochar as a humus soil enhancer. The multi-stage biorefinery would utilize coffee pulping waste during the harvest seasons and would operate between coffee harvests using other agricultural waste readily available in Colombia, such as palm rachis or sugarcane bagasse, close to the pulping operations.

**Table 2:** Composition of a mixture of 60 % pulp plus 40 % mucilage simulating coffee waste (reference: Hughes, unpublished data)

	Sucrose (mg/g)	Glucose (mg/g)	Galactose (mg/g)	Fructose (mg/g)	Xylose (mg/g)	Arabinose (mg/g)	Uronic acid (mg/g)	Fructan (mg/g)	AIR (g/kg)	Ash (g/kg)
Soluble sugars										
Pulp	4.53 ± 0.70	104.2 ± 4.77	0.46 ± 0.10	87.31 ± 4.57						
Mucilage	168.2 ± 4.97	173.7 ± 2.66	0.68 ± 0.59	167.9 ± 1.31						
Storage carbohydrates (starch)										
Pulp		5.70 ± 0.33			5.38 ± 0.32 (xyl + gal)					
Mucilage		6.24 ± 0.22			5.64 ± 0.35 (xyl + gal)					
Cell wall carbohydrates										
Pulp		110.1 ± 4.10		33.49 ± 2.25		30.95 ± 0.49	46.12 ± 1.48			
Mucilage*		33.23 ± 1.13		9.38 ± 0.48		8.64 ± 0.66	15.79 ± 0.49			
Uronic acid and fructan										
Pulp*							17.41 ± 1.72	13.29 ± 5.06		
Mucilage*							27.14 ± 0.14	35.17 ± 4.65		
Acid insoluble residue (AIR) and ash										
Pulp									130.6 ± 2.88	4.25 ± 0.97
Mucilage									42.96 ± 23.24	3.58 ± 0.64

^*^Average and standard deviation of two replicates; all other values are average and standard deviation of three replicates

### Biochemical conversion technologies

#### *Kluyveromyces* fermentation in first tank (F1)

The yeast *K. marxianus* has advantages that make it a promising candidate for use as a versatile, thermotolerant, industrial biocatalyst (Fonseca et al. 2008). It has been reported to grow at 47 °C and above (Nonklang et al. 2008) and has the ability to produce ethanol at temperatures above 40 °C (Abdel-Banat et al. 2010; Fonseca et al. 2008; Yanase et al. 2010). In addition, *K. marxianus* offers other benefits including a high growth rate and the ability to utilize a wide variety of carbohydrate substrates such as xylose, xylitol, cellobiose, lactose, arabinose, and glycerol (Nonklang et al. 2008; Rodrussamee et al. 2011). *K. marxianus* also grows on sucrose, raffinose, and inulin at 45 °C under a static condition even when glucose is present unlike *Saccharomyces cerevisiae* (Lertwattanasakul et al. 2011). Because of these advantages, *K. marxianus* is currently being developed as a viable alternative to *Saccharomyces cerevisiae* for ethanol production (Rodrussamee et al. 2011).

To improve *K. marxianus* growth and ethanol yield at elevated temperatures under microaerophilic conditions, Hughes et al. (2013) irradiated *K. marxianus* NRRL Y-1109 with UV-C. Two *K. marxianus* mutant strains survived and were isolated from the glucose plates. Both mutant strains, but not wild type, grew aerobically on glucose at 47 °C. All strains grew anaerobically at 46 °C on glucose, galactose, galacturonic acid, and pectin; however, only one strain (NRRL Y-50798) grew anaerobically on xylose at 46 °C. With glucose or galacturonic acid as carbon source, ethanol yield was higher for this strain than for wild type.

**Fig. 1:**
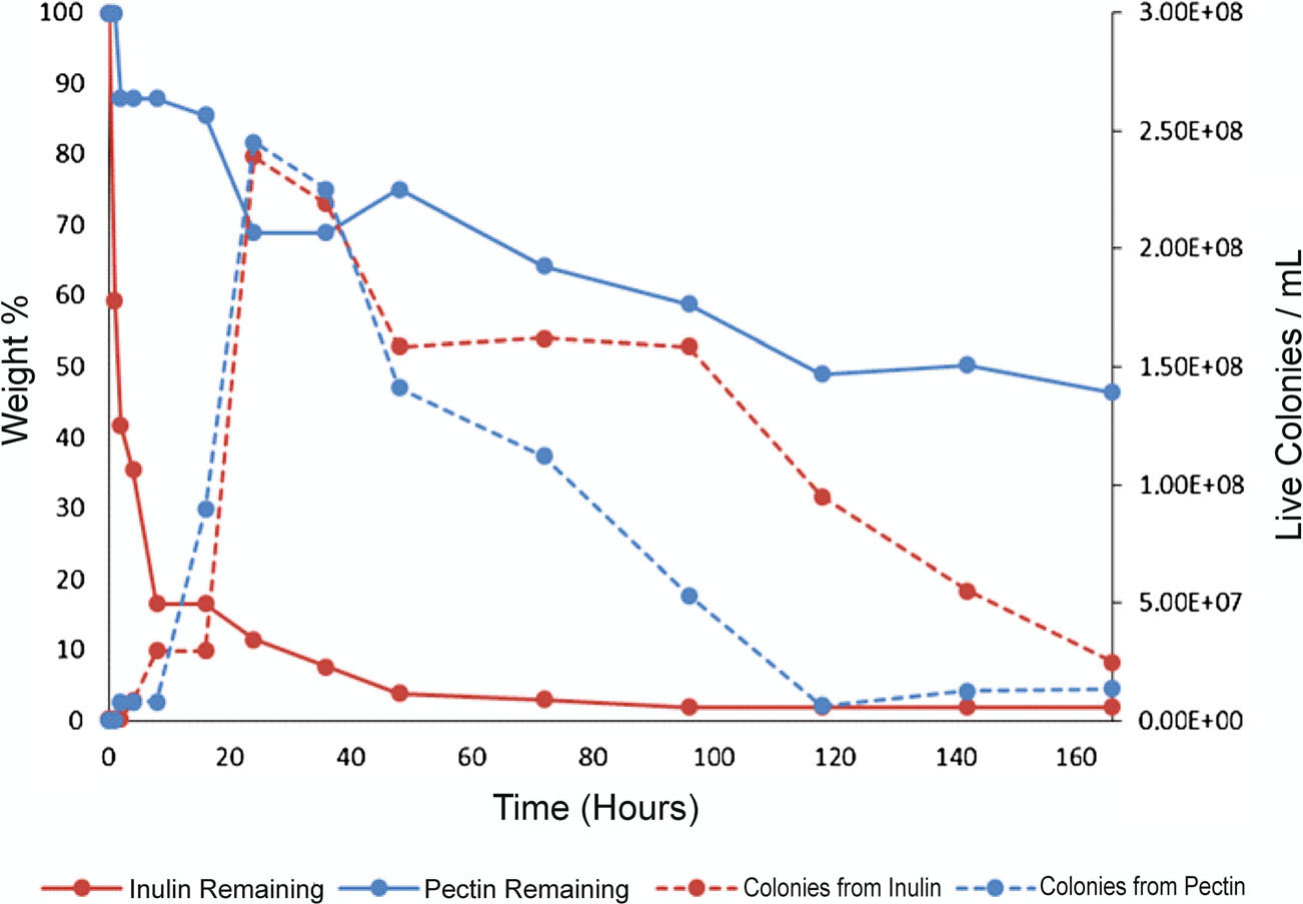
Inulin utilization and growth (live colonies/mL) of *K. marxianus* mutant strain NRRL Y-50798 in yeast–peptone–inulin (YPI; 2 %) medium and pectin utilization and cell growth of *K. marxianus* NRRL Y-50798 in coffee waste (60 % pulp plus 40 % mucilage) at 30 °C and 200 rpm over a period of 160 h. (Galindo-Leva, unpublished data)

**Fig. 2:**
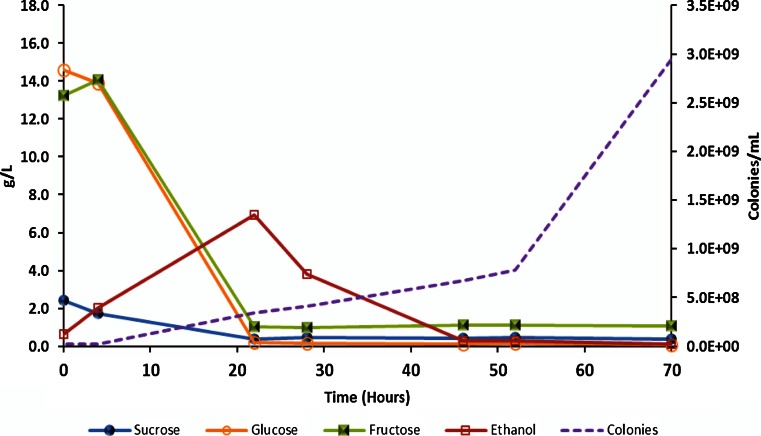
Sugar consumption, ethanol production, and cell growth of *K. marxianus* NRRL Y-50798 on coffee waste at 35 °C in a 30-L fermentor for 70 h. Sugar and ethanol concentrations determined by HPLC. (López-Núñez, unpublished data)

*Kluyveromyces marxianus* NRRL Y-50798 was tested for growth on inulin, present in garlic, onion, agave (used in tequila production), pataca, ñame, artichoke, and other products. Inulin belongs to a class of dietary fibers known as fructans and contains a high proportion of fructose monomers. *K. marxianus* NRRL Y-50798 is able to provide inulinase enzyme to convert the polymer into usable fructose (Galindo-Leva, unpublished data). Inulin utilization and growth of *K. marxianus* NRRL Y-50798 in yeast–peptone–inulin (YPI; 2 %) medium, and pectin utilization and growth of *K. marxianus* NRRL Y-50798 in coffee waste (60 % pulp plus 40 % mucilage) at 30 °C and 200 rpm for 160 h are shown in Fig. 1. The mutant strain was also tested for ethanol production from coffee waste (60 % pulp, 40 % mucilage, 12.5 % solids). The results obtained at 35 °C for sugar consumption (sucrose, glucose, fructose were measured), ethanol production and cell growth of *K. marxianus* NRRL Y-50798 using coffee waste in a 30-L fermenter for 70 h are presented in Fig. 2. The residual solid material, consisting primarily of protein, can be used directly in a fermentation stage with *Yarrowia*. The water associated with the *Kluyveromyces* fermentation can be treated using a commercial reverse osmosis desalination system to remove salts and reduce acidity.

#### *Yarrowia* fermentation in second tank (F2)

*Yarrowia lipolytica* is an oleaginous yeast species that is widely utilized in industrial applications such as production of organic acids and lipids from glucose (Papanikolaou et al. 2009). It is similar to *Escherichia coli* and *Saccharomyces cerevisiae* in ease of genetic manipulation and growth capacity. It is also able to perform post-translational processing of complex proteins, has a mainly co-translational secretion pathway, high secretion capacity and product yield, and low hyperglycosylation of products. Furthermore, production of *Yarrowia lipolytica* is relatively easy to scale up, thereby giving it advantages as a protein expression system (Gasmi et al. 2011). In addition, the whole genome of *Yarrowia lipolytica* has been sequenced (Dujon et al. 2004).

*Yarrowia lipolytica* has been the focus of studies in many research centers, and in recent years, it has been perceived as an especially attractive host for many biotechnological applications (Rywińska et al. 2013). Among the compounds produced by *Yarrowia lipolytica* are omega-3 fatty acids for use as health supplements and in the pharmaceutical, aquaculture, terrestrial animal feed, pet food, and personal care markets (Xue et al. 2013). Alpha-ketoglutaric, pyruvic, isocitric, and citric acids can be synthesized by *Yarrowia lipolytica* using n-alkanes, glucose, and glycerol as carbon sources (Finogenova et al. 2005; Papanikolaou and Aggelis 2009). Blazeck et al. (2014) undertook genotypic and phenotypic optimization of the native metabolism of *Yarrowia lipolytica* to create a strain with significantly improved lipogenesis capability. Furthermore, they demonstrated that these lipids can be readily converted into fatty acid methyl esters suitable for biodiesel. Their results support the potential of *Yarrowia lipolytica* as a platform for sustainable production of biodiesel and other important oleochemicals (Blazeck et al. 2014). The industrial potential of *Yarrowia lipolytica* is also discussed by Groenewald et al. (2014).

A recent review by Rywińska et al. (2013) provides a discussion of the characteristics of *Yarrowia lipolytica* and summarizes relevant scientific research concerning the conversion of crude glycerol discharged after the biodiesel (fatty acid methyl/ethyl esters) manufacturing process into value-added products with *Yarrowia lipolytica*. It also describes the feasibility of using *Yarrowia lipolytica* biomass, which is rich in proteins and oils, as food and feed additives and presents the different strategies employed to produce and improve yield of organic acids, such as citric, pyruvic, and α-ketoglutaric acid (Rywińska et al. 2013).

**Fig. 3:**
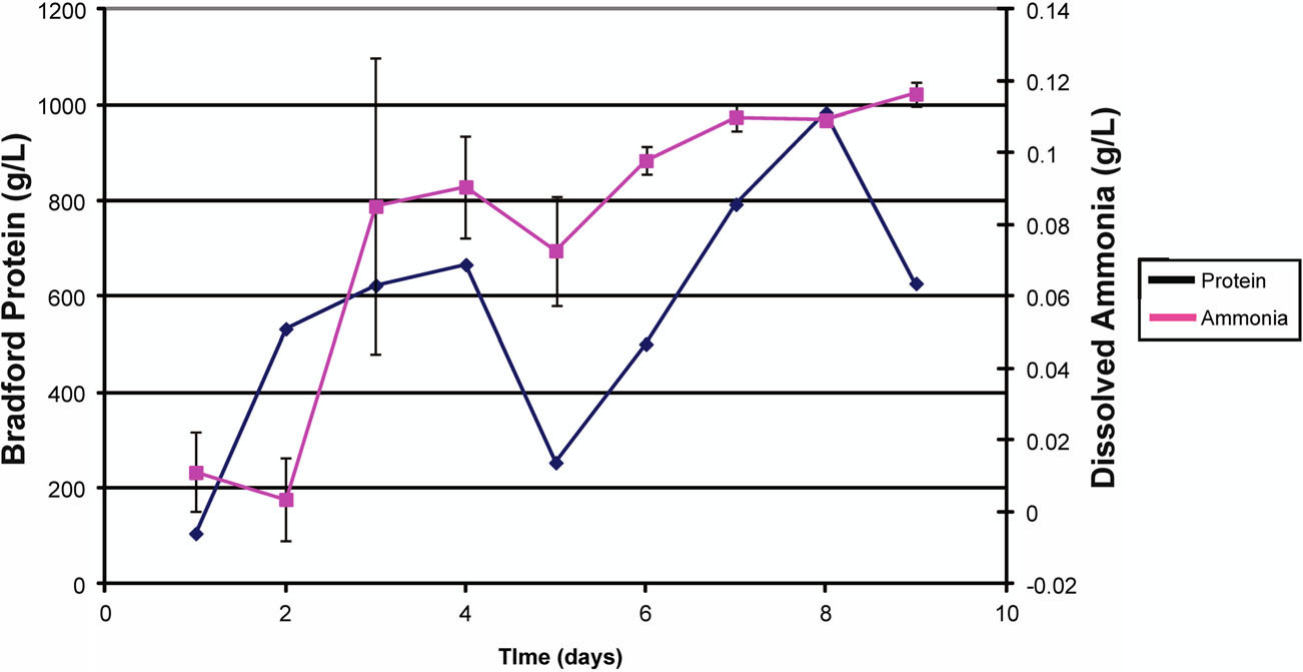
Ammonia (mean ± SD) production and protein release over a period of 7 days by *Y. lipolytica* YB-567 mutant strain F on coffee waste pretreated with *K. marxianus*. Ammonia concentration was determined using a Megazyme Ammonia Assay kit. Protein concentration was determined using the Bradford protein assay. (Hughes unpublished data)

**Fig. 4:**
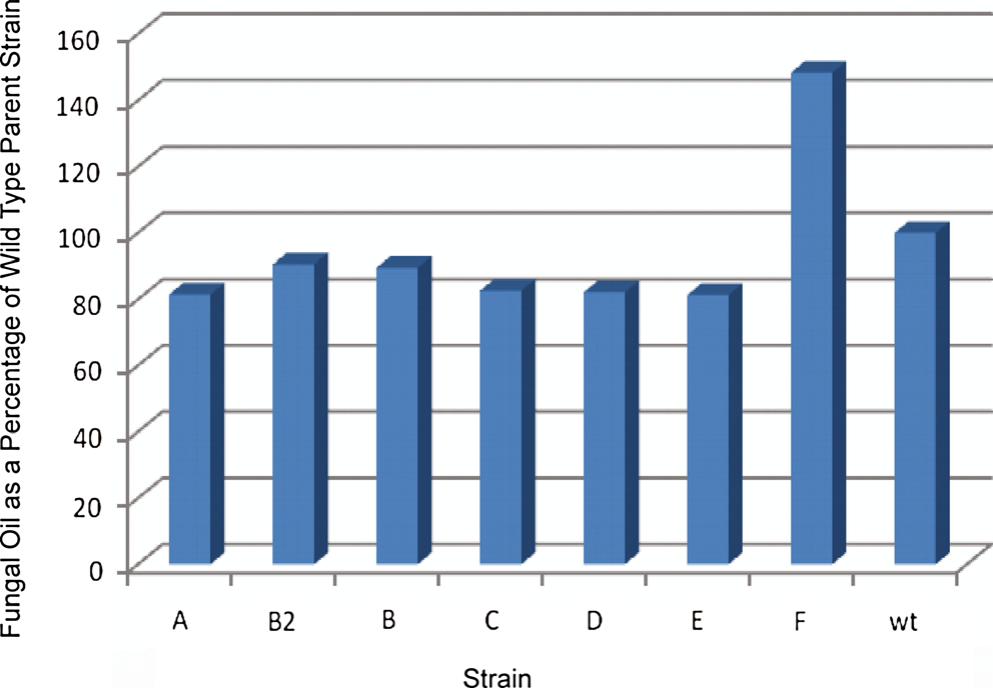
Oil production (percent by weight of production for wild type (wt) strain; wt = 100 %) for seven aerobic mutant *Y. lipolytica* strains (designated A, B2, B, C, D, E, and F) and wild type (wt) strain on coffee waste after incubation for 7 days. Concentration was determined by derivatization of oil with methanolic KOH to fatty acid methyl esters and subsequent analysis by gas chromatography. (Hughes, unpublished data)

**Fig. 5:**
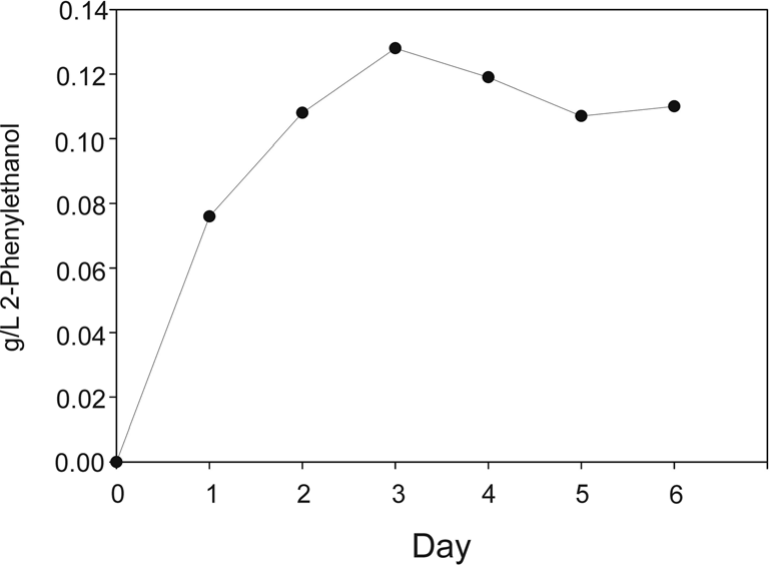
Production of 2-phenylethanol over a period of 6 days by *Y. lipolytica* mutant strain F on coffee waste pretreated with *K. marxianus*. Concentration was determined using HPLC. (Hughes, unpublished data)

In order to improve the amount of ammonia and oil produced by *Yarrowia lipolytica*, Hughes et al. irradiated *Yarrowia lipolytica* NRRL YB-567 with UV-C as described previously for *K. marxianus* NRRL Y-1109 (Hughes et al. 2013), and subsequently screened the resulting *Yarrowia* mutants for increased ammonia and triglyceride production. *Yarrowia lipolytica* NRRL Y-567 mutant strain F showed high levels of ammonia production and protein release (Fig. 3) and oil production (Fig. 4) from coffee waste. The protein is degraded by *Yarrowia lipolytica* proteases to peptides and free amino acids for animal feed. One additional commercially valuable product, phenylethanol, used as a fragrance, flavor, and antimicrobial agent (Celińska et al. 2013; Etschmann et al. 2003), is produced by *Yarrowia* at a concentration of 0.11 g/L (Fig. 5). The fatty acid profile of the oil produced by *Yarrowia* is favorable for the production of high-quality renewable gasoline and biodiesel and is presented in Table 3.

**Table 3:** Fatty acid profile of oil produced by *Y. lipolytica* mutant strain F on coffee waste pretreated with *K. marxianus* (reference: Hughes unpublished data)

Fatty acid	Without SCG (%)	With SCG (%)
C16:0 = palmitic acid	28.5	27.1
C16:1 = palmitoleic acid	1.80	8.20
C18:0 = stearic acid	30.0	20.0
C18:1 = oleic acid	3.70	2.90
C18:2 = linoleic acid	26.5	36.7
C18:3 = linolenic acid	1.35	0.92
C20:0 = arachidic acid	1.37	1.50
C22:0 = behenic acid	1.01	0.73
C30:0 = mellisic acid	0	1.22
	94.2	99.3
Monoacylglycerols (MAG)	11.0	4.70
Diacylglycerols (DAG)	37.1	33.4
Triacylglycerols (TAG)	52.0	62.0
	100.1	100.1

*SCG* spent coffee grounds (borra de café) (Mussatto et al. 2011)

#### *Saccharomyces cerevisiae* fermentation in third tank (F3)

*Saccharomyces cerevisiae* is currently the most widely employed microbial catalyst in the biotechnology industry, but this yeast does not naturally ferment xylose. Commercialization of fuel ethanol production from lignocellulosic biomass has focused on engineering the glucose-fermenting industrial yeast *Saccharomyces cerevisiae* to use pentose sugars. Extensive research efforts using innovative genetic engineering approaches have improved xylose utilization by *Saccharomyces cerevisiae* (Brat et al. 2009; Casey et al. 2013; Garcia et al. 2010; Ha et al. 2011; Hahn-Hägerdal et al. 2007; Ho et al. 1998; Hughes et al. 2009a, 2009b; Jeffries and Jin 2004; Kato et al. 2012; Kim et al. 2013; Olofsson et al. 2012; Oreb et al. 2012; Scalcinati et al. 2012; Van Maris et al. 2007; Wisselink et al. 2009), but optimization is still needed.

*Saccharomyces cerevisiae* naturally metabolizes xylulose, therefore one approach for enabling xylose utilization involves introducing the gene encoding xylose isomerase (XI), which catalyzes the direct conversion of xylose to xylulose and which may be obtained from a microorganism naturally capable of fermenting xylose. Overexpression of endogenous xylulokinase (XKS), which catalyzes the conversion of xylulose to xylulose-5-phosphate, is also necessary to overcome the naturally low expression level of this enzyme (Karhumaa et al. 2007).

Hughes et al. (2009b)) developed a three-plasmid yeast expression system utilizing the portable small ubiquitin-like modifier (SUMO) vector set combined with the efficient endogenous yeast protease Ulp1 to produce large amounts of soluble functional protein in *Saccharomyces cerevisiae*. The system consists of three vectors each with a different selectable marker (uracil (URA3), tryptophan (TRP1), or leucine (LEU2)) to provide high expression levels of three different proteins simultaneously. A PCR assembly strategy was used to clone the *Piromyces* sp. E2 XI gene into the URA-selectable SUMO vector. A library of mutagenized genes encoding a cell-penetrating peptide (Lyt-1) was cloned into the TRP-selectable SUMO vector, and either the *Yersinia pestis* XKS or transaldolase (TAL) gene was cloned into the LEU-selectable SUMO vector. All three SUMO plasmids (XI, XKS, or TAL, and Lyt-1) or the SUMO-XI plasmid alone were transformed into the INVSc1 yeast strain. Recombinant yeast strains expressing XI and XKS with or without Lyt-1 showed improved aerobic growth rates in xylose liquid medium compared to the INVSc1-XI yeast (Hughes et al. 2009b). These results demonstrated that the SUMO three-plasmid system can be used to simultaneously express high levels of several proteins that allow xylose utilization.

**Fig. 6:**
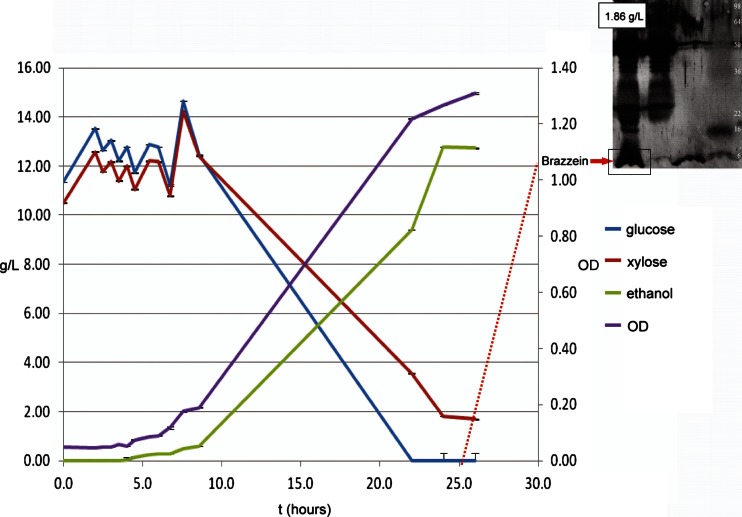
Cell biomass production (OD), sugar consumption (g/L), ethanol production (g/L), by *S. cerevisiae* NRRL Y-50183 transformed with a yeast artificial chromosome expressing brazzein a high-value co-product using 50 mL of borra (spent coffee grounds) acid/enzymatic hydrolysate in 250-mL flasks at 30 °C for 25 h. (López-Núñez unpublished data) *Inset* SDS-PAGE (12 % acrylamide gel) and Western blot analysis of immunoprecipitated brazzein expressed from this yeast artificial chromosome transformed into *S. cerevisiae* PJ69-4 diploid strain (*Lane 1 from left*, 6.5 kDa); *Lane 2* untransformed control strain; *Lane 3* Immunoprecipitated purified synthetic brazzein; Molecular markers: See Blue Plus2 ladder (Pinkelman et al., manuscript submitted)

In another study, Hughes et al. (manuscript submitted) used the SUMO fusion protein expression system to construct a yeast artificial chromosome (YAC) containing a SUMO-XI-XKS polyprotein gene for stable transformation into *Saccharomyces cerevisiae* yeast and demonstrated the feasibility of the YAC4 as a stable protein expression system in *Saccharomyces cerevisiae*. The *Piromyces* sp. E2 XI gene and the *Yersinia pestis* XKS gene were obtained by PCR amplification from the plasmids used previously (Hughes et al. 2009b) and placed into a SUMO-XI-XKS polyprotein expression cassette behind the *TRP1* promoter. The SUMO system can also be used for the concomitant expression of a value-added protein co-product, such as an insecticidal peptide (Hughes et al. 2008), the enzyme *Candida antarctica* lipase B (Hughes et al. 2011), or the natural peptide sweetener, brazzein (Pinkelman, manuscript submitted). Cell biomass production, sugar consumption, and ethanol production by recombinant *Saccharomyces cerevisiae* NRRL Y-50183 grown on borra (spent coffee grounds; Mussatto et al. 2011) acid/enzymatic hydrolysate are presented in Fig. 6 (Hughes, unpublished data). The inset in Fig. 6 shows Western blot analysis of brazzein expressed from a yeast artificial chromosome transformed into *Saccharomyces cerevisiae* PJ69-4 (Pinkelman et al., manuscript submitted).

#### *Rhodotorula glutinis* fermentation in fourth tank (F4)

The residual solids including protein and lignin can be jet cooked and passed to a fermenter for conversion into isoprenes by *Rhodotorula glutinis*. Wolf et al. (1980) reported the first isolation and characterization of eukaryotes capable of growth on methane. They examined two of the methane-utilizing yeasts, identified as strains of *Sporobolomyces roseus* and *Rhodotorula glutinis*, by electron microscopy to determine if these microorganisms showed ultrastructural modifications associated with growth on methane. Their data suggested that microbodies and the catalase contained within them play a role in eukaryotic methane metabolism (Wolf et al. 1980).

Moliné et al. (2012) demonstrated that yeasts of the genera *Rhodotorula* are able to synthesize different pigments of high economic value like β-carotene, torulene, and torularhodin. However, the low production rate of pigment in these microorganisms limits its industrial application. They described strategies to obtain hyperpigmented mutants of *Rhodotorula mucilaginosa* by means of ultraviolet-B radiation, the procedures for total carotenoids extraction and quantification, and a method for identification of each pigment. Buzzini and Martini (2000) investigated the production of carotenoids by strains of *Rhodotorula glutinis* on different raw materials of agro-industrial origin (grape must, glucose syrup, beet molasses, soybean flour extract, maize flour extract). The maximum yield (5.95 mg/L of total carotenoids culture fluid, 630 μg/g dry cell weight) was obtained with a particular strain of *Rhodotorula glutinis* after a batch culture of 120 h in a substrate containing concentrated rectified grape must as the sole carbohydrate source. In all experiments, the major pigments forming carotenoids (β-carotene, torulene, torularhodin) were quantified.

Cheirsilp et al. (2011) obtained enhanced lipid production from industrial wastes with a mixed culture of oleaginous yeast *Rhodotorula glutinis* and microalga *Chlorella vulgaris*. These wastes included effluent from seafood processing plant and molasses from a sugarcane plant. In the mixed culture, the yeast grew faster and the lipid production was higher than that in the pure cultures. This could be because microalga acted as an oxygen generator for yeast, while yeast provided carbon dioxide to microalga and both carried out the production of lipids. The highest biomass of 4.63 g/L and lipid production of 2.88 g/L were obtained after 5 days of cultivation. In addition, the plant oil-like fatty acid composition of yeast and microalgal lipids suggested their high potential for use as biodiesel feedstock (Cheirsilp et al. 2011).

**Fig. 7:**
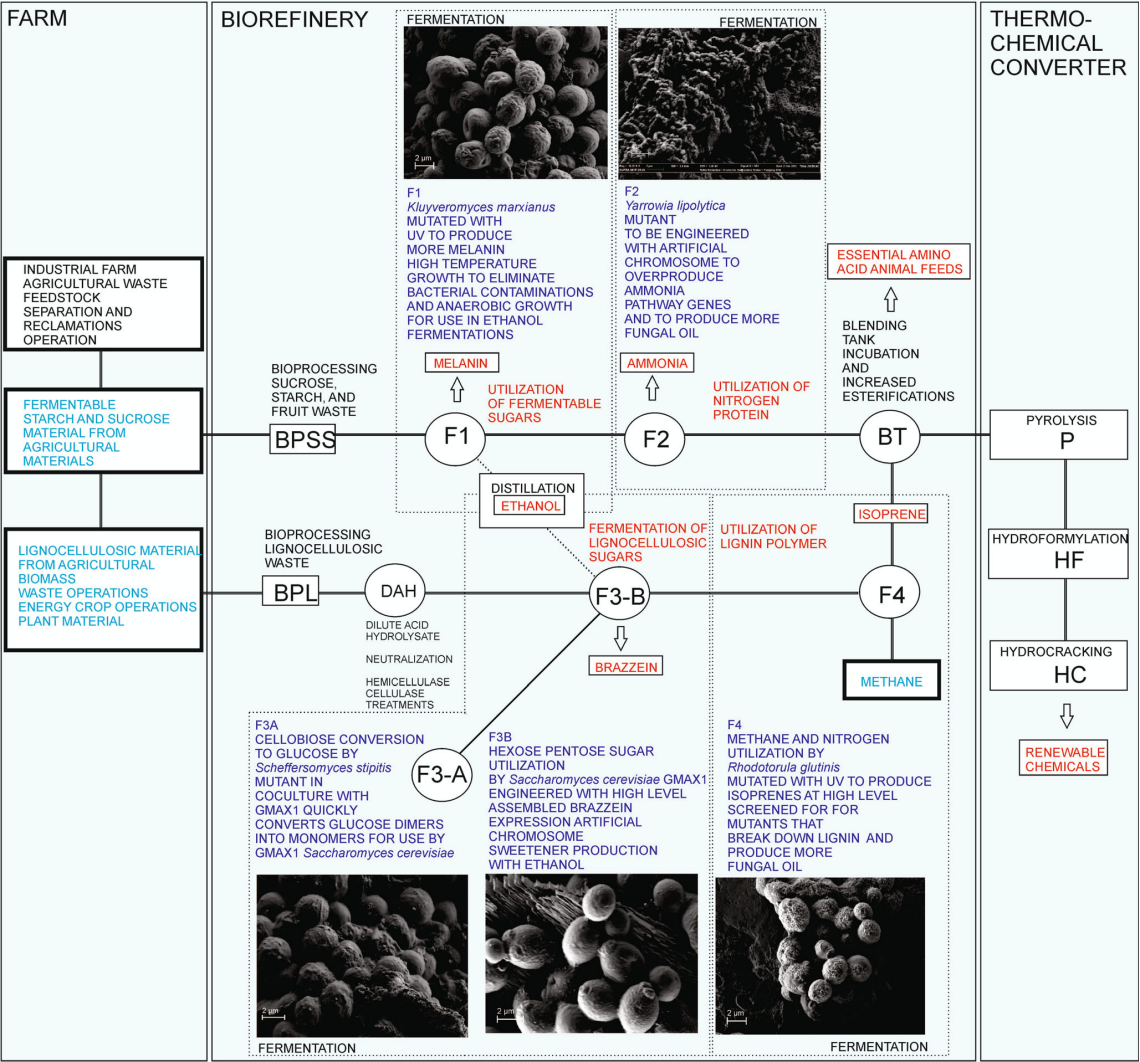
Improved strains used in the multi-stage biorefinery concept and their potential products

The fatty acid profile of the oil from *Rhodotorula glutinis* (F4) grown on residual protein and lignin solids from *Saccharomyces* tank (F3) is given in Table 4. A diagram of the improved strains proposed for use in the multi-stage biorefinery concept and the products they potentially yield are provided in Fig. 7.

**Table 4:** Fatty acid profile of the oil (tank F4) from *R. glutinis* mutant grown on residual protein and lignin solids from tanks F3 + F4 (reference: Hughes, unpublished data)

Fatty acid	Without SCG (%)	With SCG (%)
C16:0 = palmitic acid	34.3	27.1
C16:1 = palmitoleic acid	0.39	
C18:0 = stearic acid	8.08	17.0
C18:1 = oleic acid	8.26	
C18:1 = Δ^6^		6.3
C18:2 = linoleic acid	43.2	34.6
C18:3 = linolenic acid	2.73	2.37
C18:3 = Δ^9,12,15^	1.19	
C20:0 = arachidic acid		0.91
C20:1 w 5	0.26	0.24
C20:1 w 8		0.14
C20:1 w 11	0.26	0.65
C20:2 w 11, 14	0.04	1.14
C20:3 w 11,14,17		0.65
C20:4 = arachidonic	0.11	
C22:0 = behenic acid	0.63	0.57
C22:1 = erucic acid	0.14	0.30
C22:2 w 13, 16		0.49
C24:0 = lignoceric acid	0.33	0.43
C24:1		0.80
C28:0		2.31
	99.9	96.0
Monoacylglycerols (MAG)	9.0	1.2
Diacylglycerols (DAG)	32.2	26.4
Triacylglycerols (TAG)	58.8	72.4
	100	100

SCG spent coffee grounds (borra de café) (Mussatto et al. 2011)

### Thermochemical conversion technologies

#### Pyrolysis

**Fig. 8:**
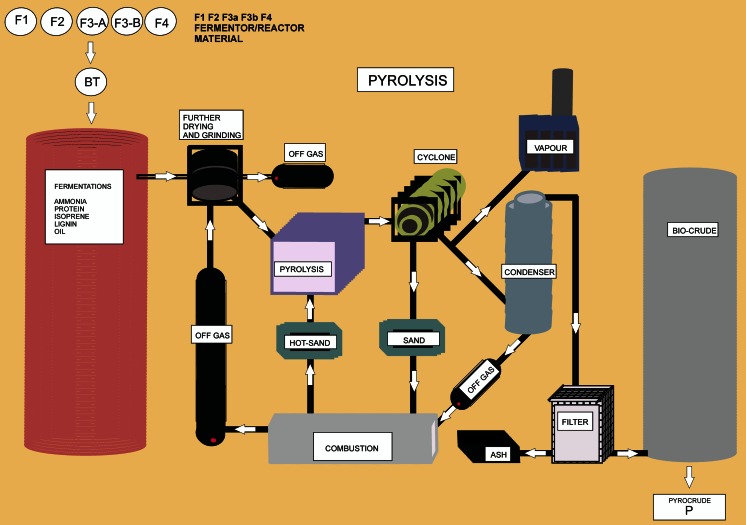
Process for pyrolysis of the residue from biochemical conversions (fermentations) for production of bio-oil (bio-crude) (Venderbosch and Prins 2010)

Pyrolysis is defined as the irreversible thermochemical decomposition of material at elevated temperature (300 + °C) in the absence of oxygen. The principal benefit of pyrolysis is conversion of low-energy density substrates into higher density liquid (bio-crude or bio-oil) and solid (biochar) fractions. A low density volatile (syngas) fraction is also produced. Pyrolysis has been utilized for centuries to produce charcoal for cooking stoves. More recently, pyrolysis is being considered for the production of transportation fuels and other products (Laird et al. 2009; Brown and Brown 2013). The distribution of pyrolysis products (among bio-oil, biochar, and syngas) is dependent on the type of pyrolysis, reaction conditions, and feedstock. Pyrolysis is classified into three categories: slow, fast, and gasification. Of these, fast pyrolysis maximizes bio-oil production, slow pyrolysis augments the yield of biochar, and gasification maximizes syngas production. With regard to production of liquid transportation fuels, fast pyrolysis is employed to produce bio-oil (Butler et al. 2011; Jarboe et al. 2011; Mohan et al. 2006; Venderbosch and Prins 2010; Yaman 2004). A review of recent laboratory research and commercial developments in fast pyrolysis and upgrading is provided by Butler et al. (2011). The properties and composition of bio-oil such as high moisture and heteroatom content, presence of oxygenates such as organic acids, and high non-oxidative reactivity prevent its direct use as a transportation fuel, thus upgrading such as hydroprocessing and distillation is necessary (Balat 2011; Butler et al. 2011; Channiwala and Parikh 2002; Elliot 2007; Mortensen et al. 2011; Pachauri and Reisinger 2007; Sorrell et al. 2010). A diagram of the process for pyrolysis of the residue from biochemical conversions (fermentations) for production of bio-oil (bio-crude) is shown in Fig. 8.

**Fig. 9:**
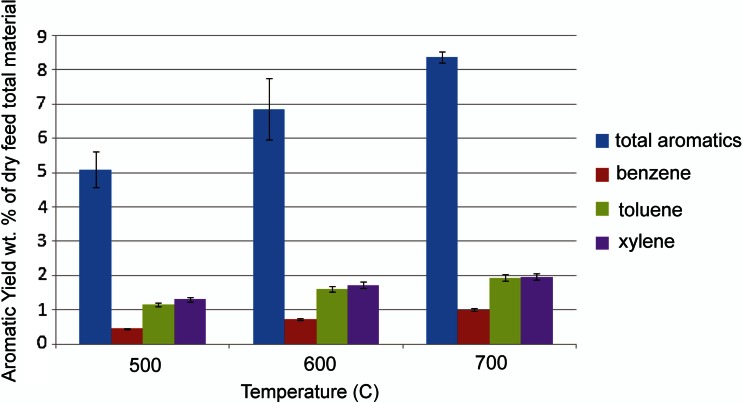
Production of aromatics by catalytic pyrolysis using HZSM-5 zeolite of the residue from coffee waste biochemical conversions. (Brown unpublished data)

Another possibility for thermally upgrading fermentation residues is catalytic pyrolysis which involves either directly mixing zeolite catalyst particles with biomass and pyrolyzing the mixture or placing the catalyst in a fixed bed immediately downstream of the pyrolysis reactor (Carlson et al. 2009). Vapors from the pyrolyzing biomass are absorbed in the pores of the zeolite where cracking and rearrangement reactions produce olefins and aromatic compounds (Carlson et al. 2011). The process is attractive in that it converts oxygenated compounds into hydrocarbons without the addition of hydrogen. Interestingly, protein is also converted into hydrocarbons with nitrogen converted mainly to ammonia. However, the catalyst readily cokes, especially when the feedstock contains large quantities of lignin. Figure 9 illustrates the yield of aromatics for the catalytic pyrolysis of the residue from the sequential biochemical processing of coffee bean waste, as described in this review. The aromatic yields, on the order of 5–9 wt%, are relatively modest compared to yields from carbohydrate- and protein-rich feedstocks, which can be as high as 30 wt% (Wang and Brown 2013), probably because the coffee waste after biochemical processing consists substantially of lignin or recalcitrant polysaccharides.

#### Hydrocracking, hydroformylation, and hydrotreating of bio-oil

Due to the complex chemical composition of bio-oils (bio-crude), hydrotreatment will be necessary to eliminate chemically bound oxygen and sulfur as well as saturate carbon bonds. The principal reactions that occur during hydrotreatment include hydrodeoxygenation, hydrodesulfurization, hydroformylation, and hydrogenation. Hydrotreatment generally occurs at operating pressures between 10–120 bar and temperatures in the range of 250–450 °C over metal catalysts and in the presence of hydrogen. Factors that contribute to catalyst deactivation include poisoning by nitrogen species, metal deposition (specifically alkali metals), and carbon deposition (coking). Alkenes, phenolic compounds, and organic acids have the strongest affinity for carbon deposition on catalyst surfaces due to their propensity to participate in polymerization and polycondensation chemistry at hydrotreatment reaction conditions. Coking can be partially ameliorated by proper choice of catalyst, reaction pressure and temperature (Fonseca et al. 1996; Richardson et al. 1995).

Once the bio-oil has been hydrotreated, it may be subjected to hydrocracking to facilitate the conversion of larger hydrocarbons into smaller, gasoline-range species but the process is also prone to coking. After cracking has been performed, a final distillation will be necessary to collect fractions equivalent to the boiling ranges of conventional gasoline and diesel fuel (Jones et al. 2009). Hydrocracking combines molecular weight reduction of the crude oil with an increase of the hydrogen content to give a useful products range (gasoline, middle distillate). Hydrocracking requires bifunctional catalysts, containing catalytically active hydrogenation and cracking sites, and that are generally composed of metal sulfides supported on an acidic silica–alumina or stabilized Y zeolite. More active palladium (Pd) can be used, in place of metal sulfides in catalysts destined for second stage hydrocracking reactors, after most of the sulfur in the feed has been removed (Maitlis and Haynes 2008).

**Fig. 10:**
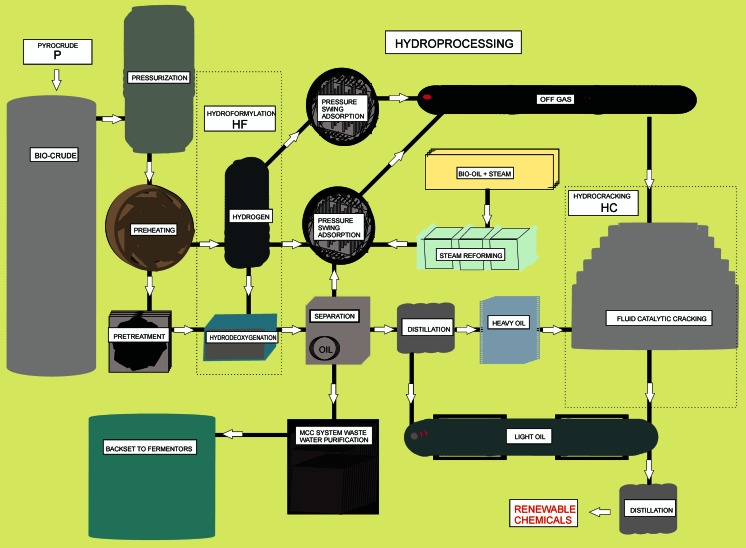
Process flow for production of renewable gas from advanced uniform fermentation streams using hydrodeoxygenation, hydroformylation, and hydrocracking. (Venderbosch and Prins 2010)

In the hydroformylation (or oxo) reaction the elements H and CHO are added to an olefin catalyzed by derivatives of dicobalt octacarbonyl. Alpha-olefins are hydroformylated to give both linear and branched chain aldehydes. The process is commercially important, especially to make C4 oxygenates (butyraldehyde and butanol) and many catalyst variations are known with considerable attention devoted to increasing their selectivity. Currently, three quarters of all industrial hydroformylation processes are based on rhodium triphosphine catalysts, especially for lower alkenes where high regioselectivity to linear aldehydes is critical (Maitlis and Haynes 2008). The process flow for production of renewable chemicals from bio-crude using hydrotreatment is outlined in Fig. 10.

Reactions of industrial interest from pyrolysis and hydrotreatment are propylene to butyraldehyde, 1-hexene to heptaldehyde, 1-octene to nonaldehyde, decene to C11 aldehyde (target being detergent alcohols), benzene from pyrocrude to produce cyclohexane, ethylbenzene to make styrene, cumene to make phenol, and acetone to make methacrylates and methyl isobutyl ketone. The significant levels of aromatic hydrocarbons as well as alkanes, such as generated by pyrolysis of the residue of biochemical conversions of coffee waste, can be used to produce several chemical feedstocks in addition to renewable gasoline and diesel fuel.

### Integration of conversion technologies for coffee and other agricultural wastes

**Fig. 11:**
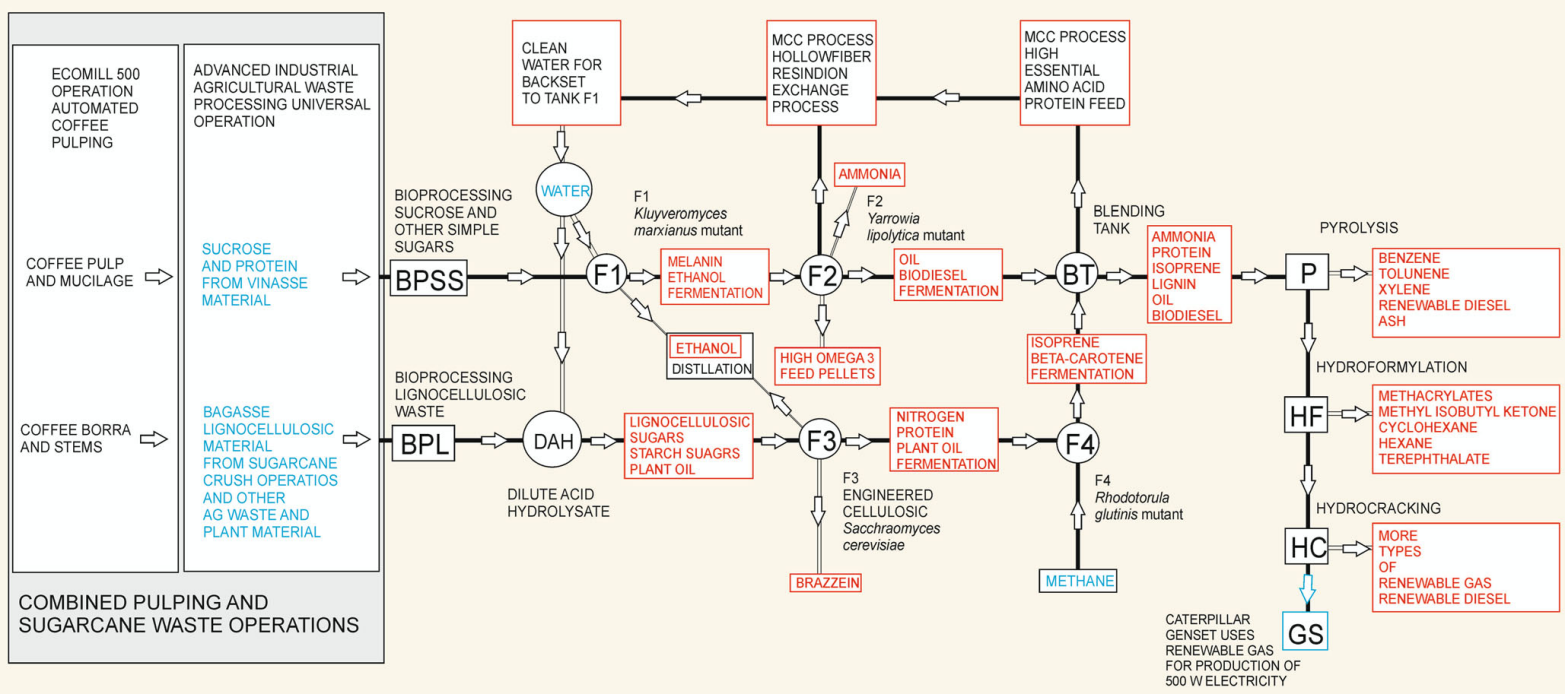
Multi-stage integrated pulping station/biorefinery concept to ensure viable continuous operation. The pulping station/biorefinery would utilize coffee pulping waste during the harvest seasons and would operate between coffee harvests using other agricultural waste readily available in Colombia, such as sugarcane, oil palm, cut flowers, bananas, rice, corn, borra (spent coffee grounds), or cocoa beans, close to the pulping operations

**Fig. 12:**
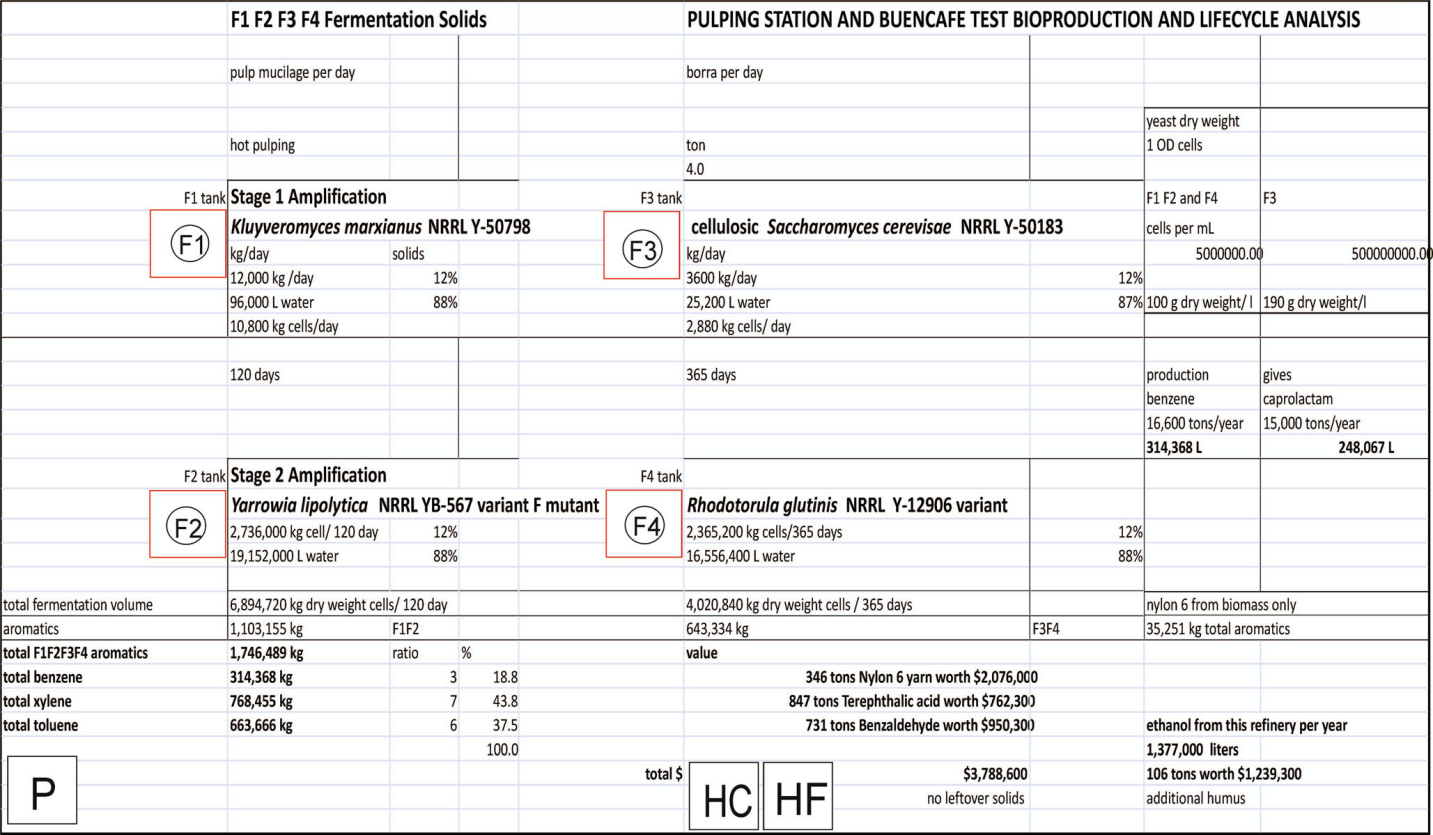
Estimate of the quantities of high-value aromatic compounds produced from a typical quantity of spent coffee grounds (borra) as feedstock in a multi-stage integrated pulping station/biorefinery. Borra is an example of a waste feedstock available year round that would supply the pulping station/biorefinery between coffee harvests

The initial stage of the proposed integrated biorefinery concept consists of fermentation processes using *Kluyveromyces* (F1), *Yarrowia* (F2), *Saccharomyces* (F3), and *Rhodotorula* (F4) for conversion of coffee waste materials to bioethanol, ammonia fertilizer, amino acids for animal feed, useful biochemicals, and oils and other organic molecules for biodiesel and for pyrolysis to produce renewable gasoline. However, harvesting coffee is not a year-round operation. To prevent down time at the biorefinery, additional stages of the biorefinery operation will process other agricultural wastes as available, primarily from sugarcane, oil palm, cut flowers, bananas, rice, corn, borra (spent coffee grounds), and cocoa beans (index mundi 2014). A diagram of the multi-stage integrated pulping station/biorefinery concept is diagrammed in Fig. 11. The pulping station/biorefinery would utilize coffee pulping waste during the harvest seasons and would operate between coffee harvests using other agricultural waste readily available in Colombia, such as sugarcane waste shown in the figure, close to the pulping operations to ensure viability of the biorefinery. As an example, the products with the greatest economic potential that would be produced by feedstock likely to be utilized at a coffee pulping station were selected to show a rough mass balance of products for a given amount of agricultural waste used as feedstock. Specifically, an estimate of the quantities of high-value aromatic compounds produced from a typical quantity of borra feedstock in a multi-stage integrated biorefinery is presented in Fig. 12.

## Perspectives

Coffee is highly important to the global economy and to the livelihood of the millions of people involved in its cultivation, processing, trading, and marketing. Coffee is Colombia’s chief agricultural export and is grown extensively throughout the country. Processing the coffee berries to produce coffee beans generates large amounts of waste materials, which have a major impact on the environment. Significant progress has been made in developing technologies to eliminate coffee waste in Colombia by converting it to useful biofuels and bioproducts. Considerable work is still required to commercialize these technologies in an integrated pulping station/biorefinery. The use of an integrated biorefinery that utilizes not only the coffee waste from the pulping stations, but also utilizes other readily available agricultural wastes that can be processed between coffee harvests, is proposed as a sustainable and profitable platform for eliminating environmental contamination from agricultural wastes in Colombia. Colombia’s environment offers a wide variety of crops distributed throughout the complex Andean coffee zone geography. The proposed design provides valuable biofuels and bioproducts and high-quality microbial biomass and oil for pyrolysis and hydroformylation. Cenicafé is currently partnering with industries and government organizations that have a stake in the economic and environmental sustainability of coffee production in Colombia to develop such platforms. The economic impact of these technologies will be substantial.
